# Characterization of the Potential Cytotoxicity of *Klebsiella pneumoniae* Isolated from Colorectal Cancer Patients against a Normal Human Fibroblast Model

**DOI:** 10.1021/acsomega.5c05845

**Published:** 2025-09-03

**Authors:** Fatma Necmiye Kaci, Cenk Daglioglu, Arzu Gormez

**Affiliations:** † 37508 Erzurum Technical University, Faculty of Science, Department of Molecular Biology and Genetics, Yakutiye, Erzurum 25050, Turkey; ‡ University of Leeds, Faculty of Medicine and Health, St. James’s University Hospital, Leeds LS2 9JT, U.K.; § 425675 Istanbul University-Cerrahpasa, Cerrahpasa Faculty of Medicine, Department of Medical Biochemistry, Fatih, Istanbul 34098, Turkey; ∥ BioMed X GmbH, Im Neuenheimer Feld 515, Heidelberg 69120, Germany; ⊥ Dokuz Eylul University, Faculty of Science, Department of Biology, Buca, Izmir 35390, Turkey

## Abstract

Colorectal cancer is one of the most common types of cancer worldwide, with a multifactorial digestive pathology. Evidence has suggested that gut microbial dysbiosis is connected to the development of colorectal cancer by generating cancer cell-conducive microenvironments. Nevertheless, the relationship between colorectal cancer pathogenesis and microorganisms has not been fully clarified to date. Here, we addressed this issue and determined the cancer-causing potential of the culture filtrate and proteins of *Klebsiella pneumoniae* on healthy cells. In this study, the culture filtrate and total proteins of *K. pneumoniae* isolated from patients with colorectal cancer were investigated to determine their cytotoxic effects against the normal human fibroblast PCS-201-012 cell model. As a result of the isolation procedure, three different *K. pneumoniae* strains (named *Kp1*, *Kp2*, and *Kp3*) were obtained from biopsy samples. Their 16S rRNA gene sequences were submitted to the GenBank database under the accession numbers MK156319, MK156320, and MK156321, respectively. The WST-8 and hemolysis tests were performed to examine the exacerbating effect of these strains on normal cells. The apoptosis-inducing ability of the isolated strains was characterized based on a combination of several techniques: determination of caspase-3 activity, JC-1 mitochondrial assay, and flow cytometry-based FITC-Annexin-V/PI double staining. Moreover, the expression profiles of four candidate genes (APC, SMAD, KRAS, TP53), which play important roles in the development of colorectal cancer, were analyzed by the qRT-PCR method. Cell biology experiments demonstrated that the culture filtrate and proteins of the related strains clearly cause cell death in normal human fibroblasts due to increased inflammatory response and necrosis. Furthermore, the culture filtrates and proteins led to a decrease in the expression of tumor suppressor genes *TP53*, *SMAD*, and *APC* and an increase in the expression of the *KRAS* oncogene, emphasizing the tumorigenicity of the strains in colorectal cancer. These results revealed that *K. pneumoniae* strains are capable of triggering cytotoxicity in normal human fibroblast cells.

## Graphical abstract

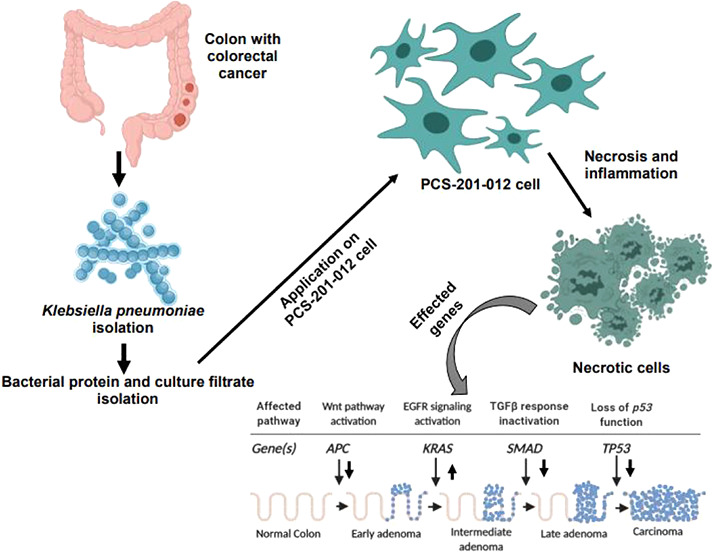

## 1 Introduction

Cancer is a critical threat throughout the globe. Several biotic and abiotic factors are associated with the growth and development of different types of cancers.^1–4^ Among these factors, bacteria play a significant role in the growth and development of different types of cancer such as lung, bladder, prostate, and colorectal cancer (CRC).^5–8^ CRC is predominantly epithelial-derived tumors, which present a broad spectrum of neoplasms ranging from benign tumors to invasive cancer. The majority of 80% of CRC cases are classified as sporadic and are associated with lifestyle factors, whereas 20% of CRC cases have a hereditary component, particularly due to specific genetic mutations that increase susceptibility to CRC and other malignancies. On the other hand, although idiopathic inflammatory diseases of the colon such as ulcerative colitis, Crohn’s disease, and celiac disease are associated with the development of small bowel or colon lymphomas, inflammatory bowel diseases (IBDs) are most strongly associated with the development of CRC.^9^

Chronic inflammation is known to account for about one-fifth of all human cancers resulting from environmental damage, diet, hereditary gene polymorphisms, infections, and dysfunction of the immune system, or combinations. Long-term intestinal inflammation has been shown to be associated with CRC and small bowel adenocarcinomas, as well as lymphoma and autoimmune diseases.^10^ The first step in the inflammation-mediated pathogenesis of CRC is chronic inflammation and regeneration. Continuity in regenerative activity causes hyperplastic tissue formation, followed by neoplastic tissue formation and benign adenoma formation.^11^

Approximately 100 trillion microorganisms (bacteria, viruses, and fungi) live in the human intestinal microbiota. The number and species of these microorganisms indicate personal differences depending on lifestyle, diet, and the host organism.^12^

It has been shown that the risk of developing cancer increases if a microorganism infection is intense in certain tissues, and these studies have confirmed that the formation of CRC affects the structure of the intestinal microbiological community.^13^ *Klebsiella pneumoniae* is a Gram-negative bacterium that can lead to a wide range of infections in humans. These infections are known to be associated with asymptomatic diverticulitis,^14^ inflammatory bowel disease,^15^ tubulovillous adenoma,^16^ CRC,^17^ and colon polypectomy.^18^

In this study, to further characterize the tumorigenicity of *K. pneumoniae* in colorectal cancer, the relationship between the strains and CRC was investigated. For this purpose, the strains were first isolated from cancerous tissues of patients with CRC. After that, 16S rRNA gene sequencing was used to identify the related organisms. In order to evaluate the cytotoxicity of the strain culture filtrates and total proteins on PCS-201-012 cells and human red blood cells (h-RBCs), WST-8 assay and hemolysis tests were performed, respectively. Caspase-3, JC-1, and Annexin-V analyses were also employed to investigate multiple cellular events in apoptosis. Moreover, the expression profiles of four candidate genes (APC, SMAD, KRAS, TP53) were analyzed by the qRT-PCR method to explore genetic alterations in healthy cells.

## 2 Materials and Methods

### 2.1 Isolation of Intestinal Bacterial Flora

In this study, bacterial isolates were obtained from biopsy specimens from cancerous tissues of 10 patients with CRC, submitted to Ataturk University Medical Faculty Hospital, Department of Pathology, during a one-year time frame (Ataturk University, Faculty of Medicine, Clinical Research Ethics Committee approval number: B.30.2.ATA.0.01.00/85) (Table 1). The patient specimens were immediately transported in an ice pack to a microbiology laboratory and processed within 24 h of collection for the bacteriological study. The protocol described in Swidsinski et al. was followed for bacterial isolation from biopsy samples.^19^ Colonies with different morphological appearances were picked aseptically from Tryptic Soy Agar (TSA) and cultured again on Columbia Blood Agar Base containing laked horse blood (Thermo), trimethoprim, cefsulodin, vancomycin, and amphotericin B.

**Table 1:** Counts of Patient Samples by Gender and Age Group

Characterstics	CRC
Gender	
Female	5
Male	5
Average Age (range)	45 (35–68)

### 2.2 Molecular Characterization of Bacteria

Genomic DNA isolation was performed for the molecular identification of bacteria. Bacteria were grown in a Columbia Blood Agar Base. After the incubation period, the viable colonies were collected and then washed with PBS and centrifuged. The supernatant was discarded, and DNA was isolated from the pellet according to the kit protocol (PureLink Genomic DNA Mini Kit, Invitrogen).

### 2.3 PCR Amplification Assays

To amplify the 16S rRNA gene region of DNA samples, universal primers targeting the bacterial 16S rRNA gene were used: 27F (5′-AGAGTTTGATCCTGGCTCAG-3′) and 1391R (5′-GACGGGCGGTGTGTRCA-3′), which bind approximately at positions 27 and 1391 of the *E. coli* 16S rRNA gene, respectively. Amplification reactions were carried out in a 50 μL volume containing 10× PCR buffer (Applied Biosystems, Roche, California, USA), 50 μM magnesium chloride, 10 mM deoxynucleoside triphosphate mix, 50 pmol of each primer, and 5 units/μL Taq DNA polymerase (Applied Biosystems). The PCR was performed under the following conditions: 1 cycle of 95 °C for 2 min, 35 cycles of 94 °C for 1 min, 53 °C for 1 min and 72 °C for 90 s, and 1 cycle of 72 °C for 10 min. 1% agarose gel was prepared for visualization of PCR products by gel electrophoresis.^20^ After PCR amplification, the PureLink PCR Purification Kit (Invitrogen) protocol was followed for the purification of the PCR amplicons.

### 2.4 16S rRNA Sequence Analysis

The sequences were aligned and uniquely identified using the BioEdit and Clustal W programs (within BioEdit). By using the NCBI (National Center for Biotechnology Information )/BLAST database, the sequences were compared to the compilation of 16S rRNA gene sequences available in the databases to determine the highest similarity to the GenBank and EMBL database sequences.

### 2.5 Bacterial Culture Filtrates

Bacterial isolates were inoculated into tryptic soy broth (TSB), prepared in a volume of 100 mL, and incubated at 37 °C under microaerophilic conditions for 24 h. At the end of the incubation period, the samples were centrifuged at maximum speed for 15 min. Then, the supernatant was passed through a 0.22 μm filter to obtain a cell-free supernatant and used as the culture filtrate.^21^

### 2.6 Bacterial Protein Isolation

Bacterial protein isolation was carried out using a modified version of the Sheykhian method.^22^ For this purpose, 1 g of bacterial cells was washed three times with cold phosphate-buffered saline (PBS). The resulting bacterial pellet was resuspended in 10 mL of Tris-HCl buffer (0.050 M, pH 7.8) containing EDTA (1 mM/L) and phenylmethylsulfonyl fluoride (PMSF) (1 mM/L). The bacterial suspension was sonicated eight times for 45 s, with 2 min intervals, using a Soniprep 150 (MSE) sonicator. After sonication, the disrupted cells were centrifuged at 1500 × *g* for 15 min. The pellet was resuspended in 10 mL of Tris-HCl buffer (0.050 M, pH 7.8), and DNase and RNase (1 mg/mL each, Sigma-Aldrich, USA) were added. The suspension was incubated at 37 °C for 2 h. Following incubation, it was centrifuged at 150,000 × *g* for 45 min at 4 °C. The resulting pellet was resuspended in 10 mL of 2% sardine (Sigma-Aldrich, USA) and incubated at room temperature for 30 min. After the supernatant was removed, the pellet was gently washed with PBS and resuspended in 1 mL of PBS (pH 7.8) containing 1 mM PMSF. Protein concentration was determined using the Bradford assay.

### 2.7 Analysis of Bacterial Proteins by SDS-PAGE

Sodium dodecyl sulfate polyacrylamide gel electrophoresis was used for the analysis of total proteins. 15 μL of the bacterial protein was mixed with 5 μL of dye and denatured at 100 °C for 5 min. The mixture was then separated using 5–15% SDS-PAGE at 75 V for approximately 3–4 h. Coomassie Blue G-250 was used for staining the proteins in the gel.

### 2.8 Cell Line and Culture Conditions

Normal adult human dermal fibroblasts (PCS-201-012) were purchased from ATCC (American Type Culture Collection, Manassas, VA, USA). The cells were grown and maintained in Dulbecco’s Modified Eagle Medium (DMEM) supplemented with 10% fetal bovine serum (FBS) and 1% penicillin–streptomycin. Cells were cultured under a humidified atmosphere at 37 °C in 5% CO_2_.

### 2.9 Analysis of Cell Viability by WST-8 Assay

The cytotoxicity of the culture filtrate and isolated proteins against PCS-201-012 cells was investigated by the Cell Viability Detection Kit-8 (CVDK-8, Ecotech Biotechnology, Turkey) based on WST-8 [2-(2-methoxy-4-nitrophenyl)-3-(4-nitrophenyl)-5-(2,4-disulfophenyl)-2*H*-tetrazolium, monosodium salt] quantification. PCS-201-012 cells were incubated in 96-well plates with 2 × 10^4^ cells in each well and grown overnight. The cells were then incubated with increasing concentrations of the culture filtrates [6.25% (1:15 dilution); 12.5% (1:7 dilution); 25% (1:3 dilution); 50% (1:1 dilution); and 100% (undiluted)] and isolated proteins (31.25, 62.5, 125, and 250 μg/mL) for 48 h at 37 °C under 5% CO_2_.

Following this incubation, 10% WST-8 solution was added to each well under sterile and dark conditions, and cultures were incubated at 37 °C in 5% CO_2_ for 3–4 h. The absorbance of the 96-well plate was measured at a 450 nm wavelength in a spectrophotometer to determine the number of viable cells. The absorbance of dissolved formazan in the visible region correlates with the number of viable cells.^23^ IC_50_ values were calculated from dose–response curves (protein concentration vs cell survival fraction) obtained in repeat experiments.

### 2.10 Determination of Hemolytic Activity

Hemolysis tests were performed to evaluate the effects of the culture filtrates and isolated proteins on human red blood cells (h-RBCs). The protocol described by Wang et al. was followed to determine hemolytic activity.^24^

### 2.11 Detection of Caspase-3 Enzyme Activity

Changes in caspase-3 enzyme activity in the cells are an important sign of apoptosis. To assess the effect of the culture filtrates and isolated proteins on cell death, caspase-3 activity was analyzed fluorometrically by means of the Caspase-3/CPP32 Colorimetric Assay Kit according to the manufacturer’s instructions (BioVision Research Products, USA). For this purpose, PCS-201-012 cells (1 × 10^6^) were treated with the culture filtrate (50% and 100%) and the IC_50_ concentration of isolated proteins for 48 h at 37 °C under 5% CO_2_. This method is based on the hydrolysis of the peptide substrate *N*-acetyl-Asp-Glu-Val Asp-*p*-nitroanilide by caspase-3, resulting in the release of the *p*-nitroaniline moiety. *p*-Nitroaniline was then measured at 405 nm using a plate reader.^25^

### 2.12 Detection of the Loss of Mitochondrial Membrane Potential

One of the important markers for confirming caspase-3 enzyme activity is the determination of the decrease in mitochondrial enzyme activity. For this purpose, a JC-1 (5,5′,6,6′-tetrachloro-1,1′,3,3′-tetraethylbenzimidazolcarbocyanine iodide) Mitochondrial Membrane Potential Assay Kit (Cayman) was used. JC-1 dye accumulates in mitochondria in a potential-dependent manner, shifting its fluorescence emission from red to green when the mitochondrial membrane potential is disrupted. Experimental steps were performed in accordance with the kit procedure. Briefly, PCS-201-012 cells were seeded into 6-well plates at a density of 1 × 10^6^ cells per 2 mL per well and treated with the culture filtrates (50% and 100%) and IC_50_ concentration of isolated proteins. After 48 h of incubation, the cells were collected and centrifuged at 1000 rpm for 10 min. The pellet was dissolved in 300 μL of full medium, and subsequently, 30 μL of JC-1 dye was added to each sample. The cells were incubated for approximately 30 min at 37 °C, followed by centrifugation at 400 × *g* for 5 min. After centrifugation, the pellet was dissolved in 300 μL of assay buffer, and 100 μL of each sample was transferred to black 96-well plates to measure fluorescence. A spectrophotometer was used to measure the green fluorescence at 485 and 535 nm and the red fluorescence at 535 and 595 nm. Ratios of green/red fluorescence were used to analyze the apoptosis rate.^26^

### 2.13 Detection of the Apoptotic Cell Population by Annexin-V Assay

To investigate the apoptosis-inducing ability of the culture filtrates and proteins, in addition to Caspase-3 enzyme activity changes and mitochondrial membrane potential loss, the percentage of cells undergoing apoptosis was analyzed by flow cytometry with FITC-Annexin-V/PI double staining. This was done with the Annexin-V Apoptosis Detection Kit (Beckman Coulter) according to the manufacturer’s instructions. PCS-201-012 cells were seeded in 6-well plates at a density of 1 × 10^6^ cells/2 mL per well and treated with the culture filtrates (50% and 100%) and the IC_50_ concentration of isolated proteins. After 48 h of incubation, cells were collected and centrifuged at 500 × *g* for 5 min at 4 °C, following two washes with cold PBS. The supernatant was discarded, and the resulting pellet was dissolved in cold “1× Binding Buffer.” For each sample, 100 μL of the cell suspension was transferred into glass FACS tubes placed on ice. 5 μL of propidium iodide (PI) and 1 μL of Annexin A5-FITC were added to each sample. After the samples were incubated in the dark on ice for 15 min, 400 μL of cold “1× Binding Buffer” was added to each sample, and the results were analyzed using a flow cytometer.

### 2.14 Total RNA Isolation, cDNA Synthesis, and PCR Array

To investigate changes in gene expression after the treatment and to compare them with those in the control group, RNA isolation, cDNA synthesis, and qRT-PCR assay were performed as described in our previous work.^18^ Briefly, PCS-201-012 cells were treated with the culture filtrates (50% and 100%) and the IC_50_ concentration of isolated proteins for 48 h. After incubation, RNA isolation was performed according to the PureLink RNA Mini Kit (Invitrogen, MA, USA) protocol. Following the manufacturer’s recommended procedure, the High-Capacity cDNA Reverse Transcription Kit was used for cDNA synthesis. In order to determine mRNA levels of the selected genes (*APC*, *KRAS*, *SMAD*, *TP53*, and *GAPDH*), cDNAs obtained by reverse transcription were amplified by real-time polymerase chain reaction (qRT-PCR) in the presence of specific primers. Sequences of the primers were designed using Primer3 software (http://primer3.ut.ee/) and the following primers were used for gene amplification: *APC*, 5′-CTGAGCGGCAGAATGAAG-3′ and 5′-TTGGTTCCCAGATGACTTGT-3′; *KRAS*, 5′-TTTTGTCTCCTTTCCACTGC-3′ and 5′-CCATTTCATACTGGGTCTGC-3′; *SMAD*, 5′-ATTCACGCCGCCAGTTGT-3′ and 5′-CACTTTTCTTCCTGCCCATT-3′; *TP53*, 5′-GAGGATGGGGAGTAGGACAT-3′ and 5′-ATTCAACAGTGAGGGACAGC-3′; *GAPDH*, 5′-TTCCCAAAGTCCTCCTGTTT-3′ and 5′-ATGGTGTCTGAGCGATGTG-3′ (Sentegen Biotech, Turkey). SYBR Green-based qPCR was performed with initial denaturation at 95 °C for 10 min, followed by 40 cycles of 95 °C for 15 s and 60 °C for 60 s. Melt curve analysis was conducted to verify the specificity of the PCR products. The quality of the amplified fragments was monitored using melting curve analysis and agarose gel electrophoresis. Quantitative values were obtained from the PCR quantification cycle number (Cq) in the exponential growth phase. The target mRNA abundance in each sample was normalized to the GAPDH mRNA level. All real-time PCR analyses were performed in triplicate. 2^– ΔΔCT^ method was used to calculate the differences in gene expression after qPCR analysis.^27^

### 2.15 Statistical Analysis

Statistical analysis was performed with GraphPad Prism 7.00 (GraphPad Software, Inc., La Jolla, CA, USA). The values were analyzed by using one-way ANOVA, followed by Dunnett’s multiple comparisons test where appropriate. In both tests, the differences were considered statistically significant with *p* < 0.01. A *p* value higher than 0.01 (*p* > 0.01) was accepted as not significant and defined as “ns”.

## 3 Results and Discussion

### 3.1 16S rRNA Gene Sequence Analysis of Bacteria

The 16S rRNA gene is a highly conservative component of bacterial genomes that contains regions varying between different species. Thus, 16S rRNA sequence analysis was performed for the identification of bacterial strains. Since the 16S rRNA gene is approximately 1500 base pairs long, the amplified regions of the bacterial isolates contained about 1340–1357 nucleotides (Figure 1). Then, the sequences were examined using BLAST in the GenBank database, as shown in Table 2. *K. pneumoniae* was identified at a rate of 99% from bacterial isolates, and all subsequent studies were performed with three *K. pneumoniae* strains named *Kp1*, *Kp2*, and *Kp3,* respectively.

**Table 2:** Identification Results of 16S rRNA Sequences with GenBank Databases

Bacteria strains	GenBank accession number	Identification results	SI^a^	Base pair
*Kp1*	MK156319	*Klebsiella pneumoniae*	99%	1340
*Kp2*	MK156320	*Klebsiella pneumoniae*	99%	1346
*Kp3*	MK156321	*Klebsiella pneumoniae*	99%	1357

a SI: similarity index.

**Figure 1:**
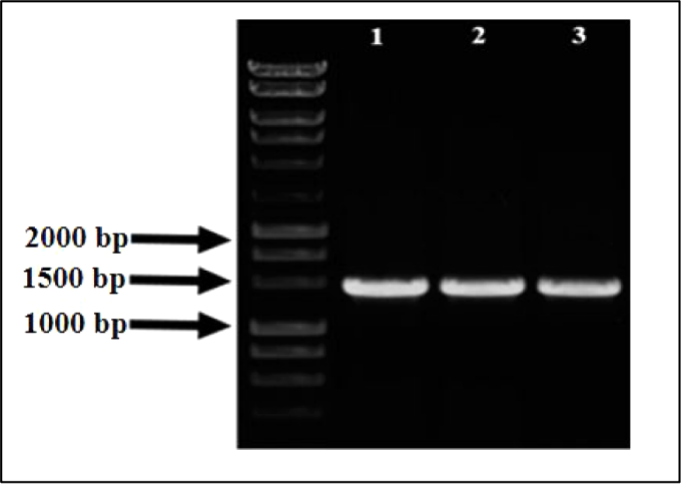
Amplification of 16S rRNA gene fragments from bacterial isolates. Agarose gel electrophoresis was used to examine the PCR products of the 16S rRNA gene. Bacterial isolates *Kp1*, *Kp2*, and *Kp3* are indicated by lanes 1–3. A molecular weight marker (leftmost lane) was a 1 kb DNA ladder. Successful amplification is confirmed by the expected band of about 1400 base pairs.

### 3.2 Bacterial Protein Profiles

The protein profiles of isolated *K. pneumoniae* strains were examined by SDS-PAGE analysis, and the results showed that the molecular masses of total proteins were in the range of 25–100 kDa, whereas the extracellular proteins exhibited protein bands between 20 and 75 kDa (Figure 2). Benedi et al. reported that the molecular weights of outer membrane proteins from different *K. pneumoniae* strains ranged from 14.5 to 92 kDa.^28^ Brinkworth et al. also demonstrated that *K. pneumoniae* grown in different media exhibited protein bands ranging from 38 to 203 kDa.^29^ While SDS-PAGE enabled general protein profiling, it lacks the resolution to identify specific virulence factors or toxins. Future studies utilizing proteomic techniques such as LC-MS/MS and targeted Western blotting are warranted to comprehensively characterize cytotoxic components in *K. pneumoniae* isolates.

**Figure 2:**
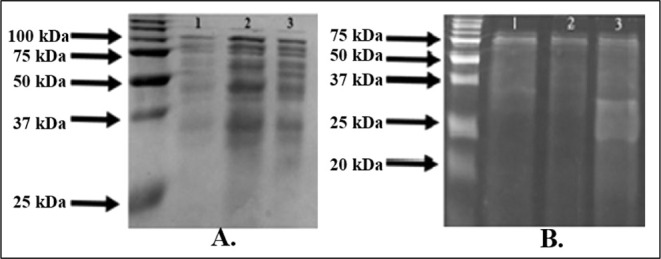
SDS-PAGE profiles of total and extracellular proteins from bacterial isolates. (A) Lanes 1–3 represent total protein profiles of *Kp1, Kp2*, and *Kp3*, respectively. (B) Lanes 1–3 show extracellular protein profiles of *Kp1, Kp2,* and *Kp3*, respectively.

### 3.3 Cytotoxic Effects of Bacterial Proteins and Culture Filtrates

To examine how the culture filtrates and isolated proteins of the strains affected healthy cell growth, the proliferation of PCS-201-012 cells was evaluated using the WST-8 cell quantification assay. PCS-201-012 cells were exposed to increasing concentrations of the culture filtrates (6.25%, 12.5%, 25%, 50%, and 100%) and isolated proteins (31.25, 62.5, 125, and 250 μg/mL). Among all proteins tested, *Kp1* and *Kp2* strains showed stronger and similar cell inhibitory effects with approximately 2.0-fold lower IC_50_ values (the half-maximal inhibitory concentration) compared to the *Kp3* strain. IC_50_ values of isolated proteins were found to be 46 ± 4.3 μg/mL, 46 ± 7.1 μg/mL, and 92 ± 5.9 μg/mL for *Kp1*, *Kp2*, and *Kp3*, respectively (Figure 3A). On the other hand, as seen in Figure 3B, the viability of the cells exposed to the culture filtrates was found to be 14.57%, 43.79%, and 22.19%, respectively, after the application of 100% culture filtrate compared to the control group for *Kp1*, *Kp2*, and *Kp3*, respectively. Therefore, the first two doses (50% and 100%) were preferred for the following experiments.

**Figure 3:**
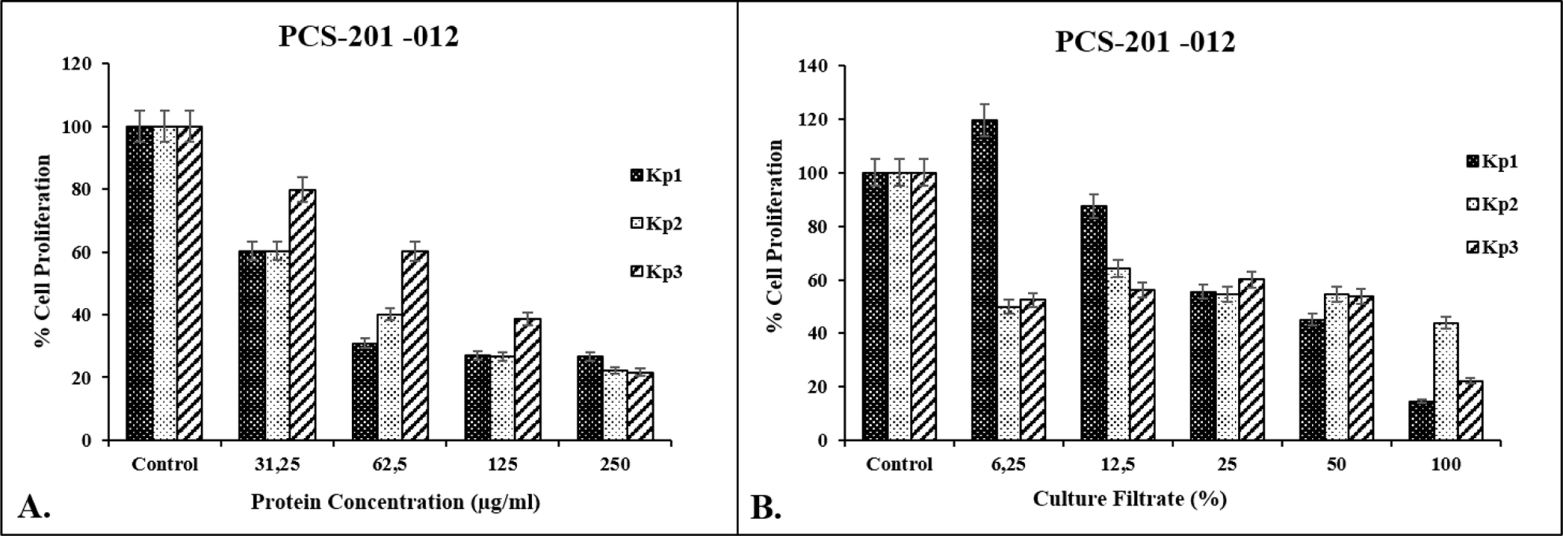
(A) Cell viability of PCS-201-012 cells after 48 h of incubation with isolated proteins (31.25–250 μg/mL). (B) Cell viability of PCS-201-012 cells after 48 h of incubation with the culture filtrates (6.25–100%). The results are expressed as a percentage of cell viability or cell number obtained in the untreated controls. Each column represents the mean ± SD of three independent experiments performed in triplicate, normalized to nontreated cells (taken as 100%). Statistical analysis was performed using GraphPad Prism 7.00. ANOVA: Dunnett’s multiple comparison test was used to calculate the values.

### 3.4 Determination of Hemolytic Activity

To assess the cytotoxic effect of isolated proteins and culture filtrates on human RBCs, the hemolytic activity was measured. Hemolytic activity tests were carried out by using commercially obtained blood samples. For the controls, 200 μL of diluted red blood cells (RBCs) were mixed with 800 μL of dH_2_O as a positive control and with 800 μL of D-PBS as a negative control. Each condition was tested in triplicate. The results showed that neither isolated proteins nor the culture supernatants from *K. pneumoniae* isolates negatively impacted mammalian red blood cells, and they did not cause hemolysis under the investigated conditions (Figure 4).

**Figure 4:**
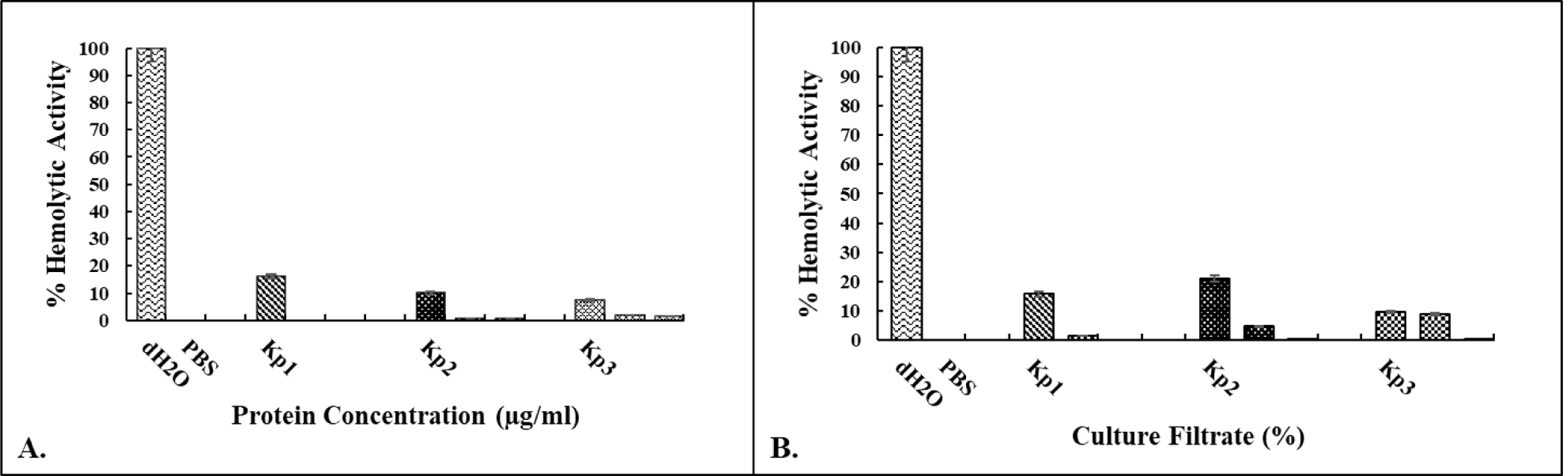
Hemolytic activity of isolated proteins and culture filtrates on red blood cells. (A) The hemolytic effects of isolated proteins from *K. pneumoniae* strains. *Kp1*, *Kp2*, and *Kp3* were evaluated at various concentrations. For *Kp1* and *Kp2*, protein concentrations of 62.5, 46, and 31.25 μg/mL were tested; for *Kp3*, protein concentrations of 125, 92, and 62.5 μg/mL were tested, respectively. Deionized water (dH_2_O) was used as the positive control (complete hemolysis) and D-PBS as the negative control (no hemolysis). (B) 100%, 50%, and 25% doses of the culture filtrates were applied to red blood cells, respectively. When compared to the positive control, none of these filtrate dilutions caused hemolysis.

### 3.5 Apoptotic Effects of Bacterial Proteins and Culture Filtrates on the Caspase-3 Enzyme Activity

Changes in caspase-3 enzyme activity of the cells are an important sign of apoptosis. Incubation of PCS-201-012 cells with the culture filtrates (100% and 50%) demonstrated considerable induction of caspase-3 activity when compared with untreated cells. 1.15- and 1.21- (*Kp1*), 1.16- and 1.04- (*Kp2*), and 1.24- and 1.21-fold (*Kp3*) increases were found for 100% and 50% concentrations of the culture filtrates, respectively (Figure 5A). However, in contrast to the culture filtrate application, we observed no increases in caspase-3 activity in the cells incubated with isolated proteins compared to untreated controls (Figure 5B). These results showed that the bacterial proteins do not induce apoptotic cells but necrotic cells as confirmed by flow cytometry. We had previously demonstrated that the absence of caspase-3 positivity is a main indicator of ongoing necrotic cell death.^30^

**Figure 5:**
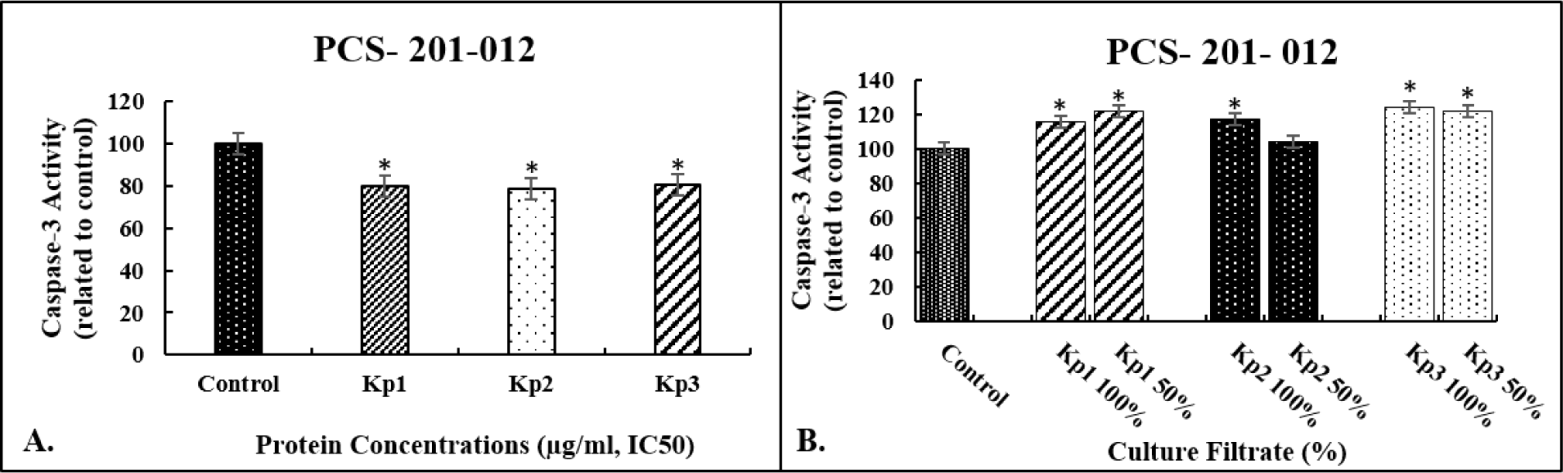
Effects of the culture filtrates (A) and isolated proteins (B) on the caspase-3 activation of PCS-201-012 cells. Caspase-3 activity was analyzed fluorometrically by means of hydrolysis of the fluorogenic substrate *N*-acetyl-Asp-Glu-Val-Asp-pNA. Each column represents the mean values (±SD) of three independent experiments normalized to nontreated cells (taken as 100%); *p* < 0.01 was considered significant, *p < 0.01 versus control.

### 3.6 Loss of Mitochondrial Membrane Potential in Response to Bacterial Proteins and Culture Filtrates

Another crucial marker in determining apoptosis is the measurement of mitochondrial damage. Loss of the mitochondrial membrane potential through changes in permeability is a step in the process of apoptosis. For this purpose, 100% and 50% doses of the culture filtrates were applied to PCS-201-012 cells for 48 h, and the results were evaluated fluorometrically. Compared to the untreated control group, when cells were treated with the 100% culture filtrate, 3.56-, 1.64-, and 1.96-fold increases were found for *Kp1*, *Kp2*, and *Kp3* strains, respectively (*p* < 0.01) (Figure 6A). On the other hand, when IC_50_ doses of isolated proteins were applied to PCS-201-012 cells for 48 h, 1.30-, 1.90-, and 1.33-fold increases were observed for *Kp1*, *Kp2*, and *Kp3* strains as compared to the control group, respectively (Figure 6B). These results showed that lower Caspase-3 activity in PCS-201-012 cells treated with the bacterial proteins led to less loss of mitochondrial membrane potential compared to the culture filtrate. This implies that the bacterial proteins induce cell death via another pathway rather than apoptosis.

**Figure 6:**
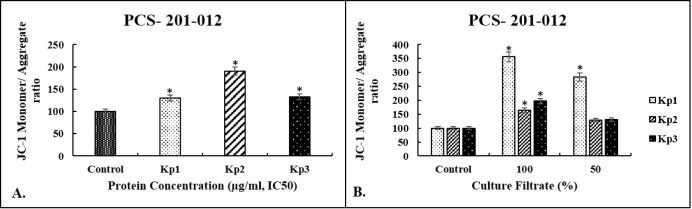
Changes in mitochondrial membrane potential in response to the bacterial proteins (A) and culture filtrates (B) on PCS-201-012 cells. Each column represents the mean ± SD of three independent experiments performed in triplicate normalized to nontreated cells (taken as 100%); *p* < 0.01 was considered significant, *p < 0.01 versus control.

### 3.7 Detection of Apoptotic Cell Population by Flow Cytometry

To examine the apoptosis-inducing ability of the culture filtrates and bacterial proteins in PCS-201-012 cells, the percentage of cells undergoing apoptosis was analyzed by flow cytometry with FITC-Annexin-V/PI double staining (Figure 7). The impact of 50% concentration of the culture filtrates and IC_50_ concentration of bacterial proteins on proapoptotic activity was investigated, and the results showed that treatment with the culture filtrates increased the number of necrotic cell populations by nearly 9.1% compared to bacterial proteins, which is in line with caspase-3 activity experiments. These results indicate that the cells did not die by apoptosis but through the necrotic pathway. The absence of certain changes in caspase-3 activity could be attributed to induced cytotoxicity, which results in rapid cell death. In parallel with these results, Yang et al. demonstrated that infected HepG2 cells with a strain of highly virulent *Kp* isolated from mice undergo apoptosis early after infection and progress to necrosis.^31^

**Figure 7:**
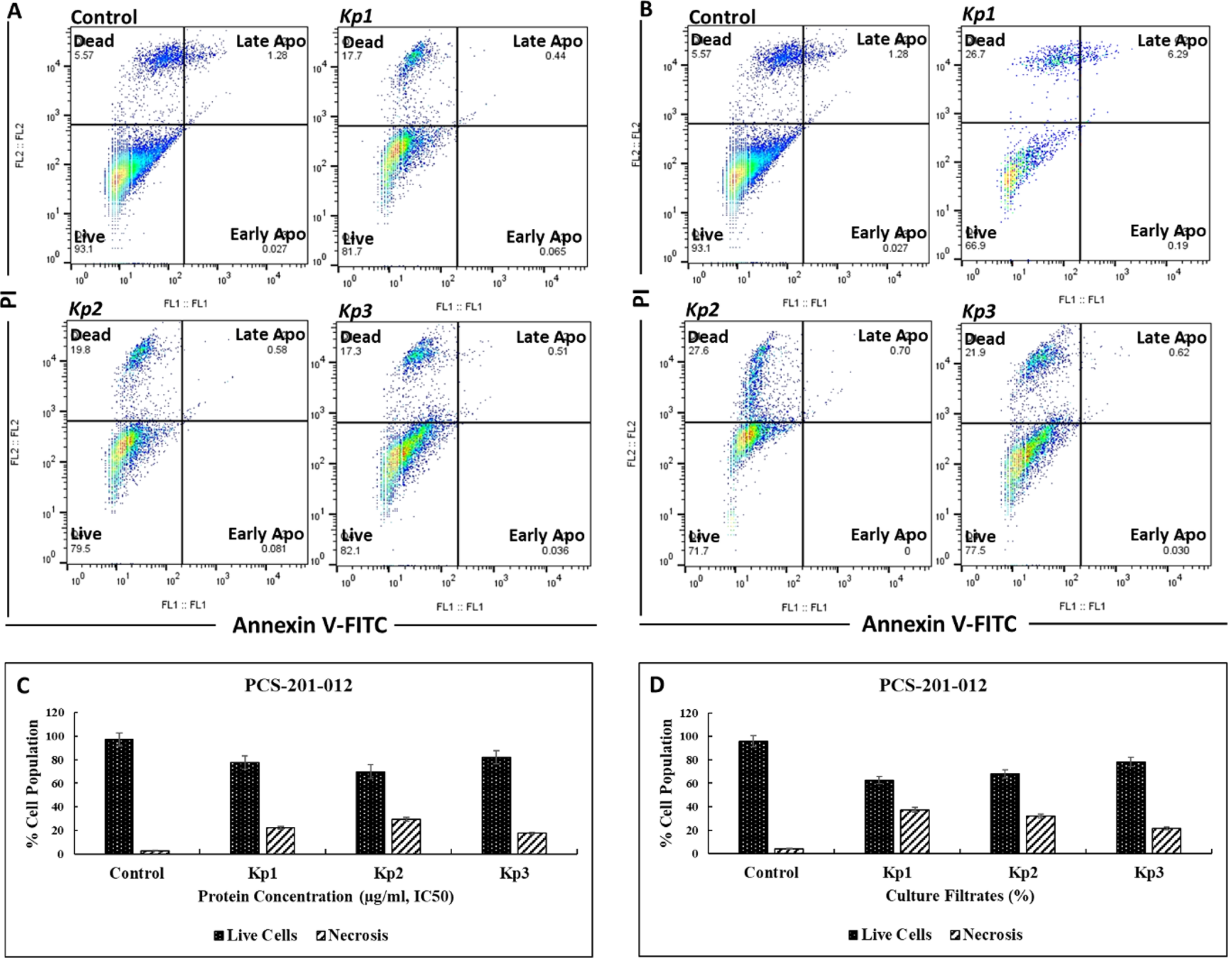
Representative images of apoptosis induction by (A) the bacterial proteins and (B) culture filtrates in PCS-201-012 cells, measured by flow cytometry. Flow cytometric plots illustrate viable cells labeled Annexin V-FITC(−)/PI(−), early apoptotic cells labeled Annexin V-FITC­(+)/PI(−), and late apoptotic cells labeled Annexin V-FITC­(+)/PI­(+). Nontreated cells were used as a control. The phase composition percentage of PCS-201-012 cells treated with (C) the bacterial proteins and (D) culture filtrates. Nontreated cells were used as a control. Data are presented as the mean ± SD from three independent experiments conducted in triplicate (*n* = 3).

### 3.8 Changes in Gene Expression Levels in Response to Bacterial Proteins and Culture Filtrates

In this experiment, the expression profiles of four candidate genes (*APC*, *SMAD*, *KRAS*, *TP53*) were analyzed by qRT-PCR to explore genetic alterations in PCS-201-012 cells after the culture filtrates and bacterial proteins incubation. Those four candidate genes were selected due to their important roles in the development of CRC. PCS-201-012 cells were treated with an IC_50_ concentration of isolated proteins and culture filtrates (50%). The results showed that both the culture filtrates and bacterial proteins downregulated the tumor suppressor *APC*, *SMAD*, and *TP53* genes, whereas they upregulated the oncogene *KRAS* gene in PCS-201-012 cells (Figure 8). Compared to the bacterial proteins, the culture filtrates exhibited more tumorigenic potential in the modulation of the gene expression of PCS-201-012 cells.

**Figure 8:**
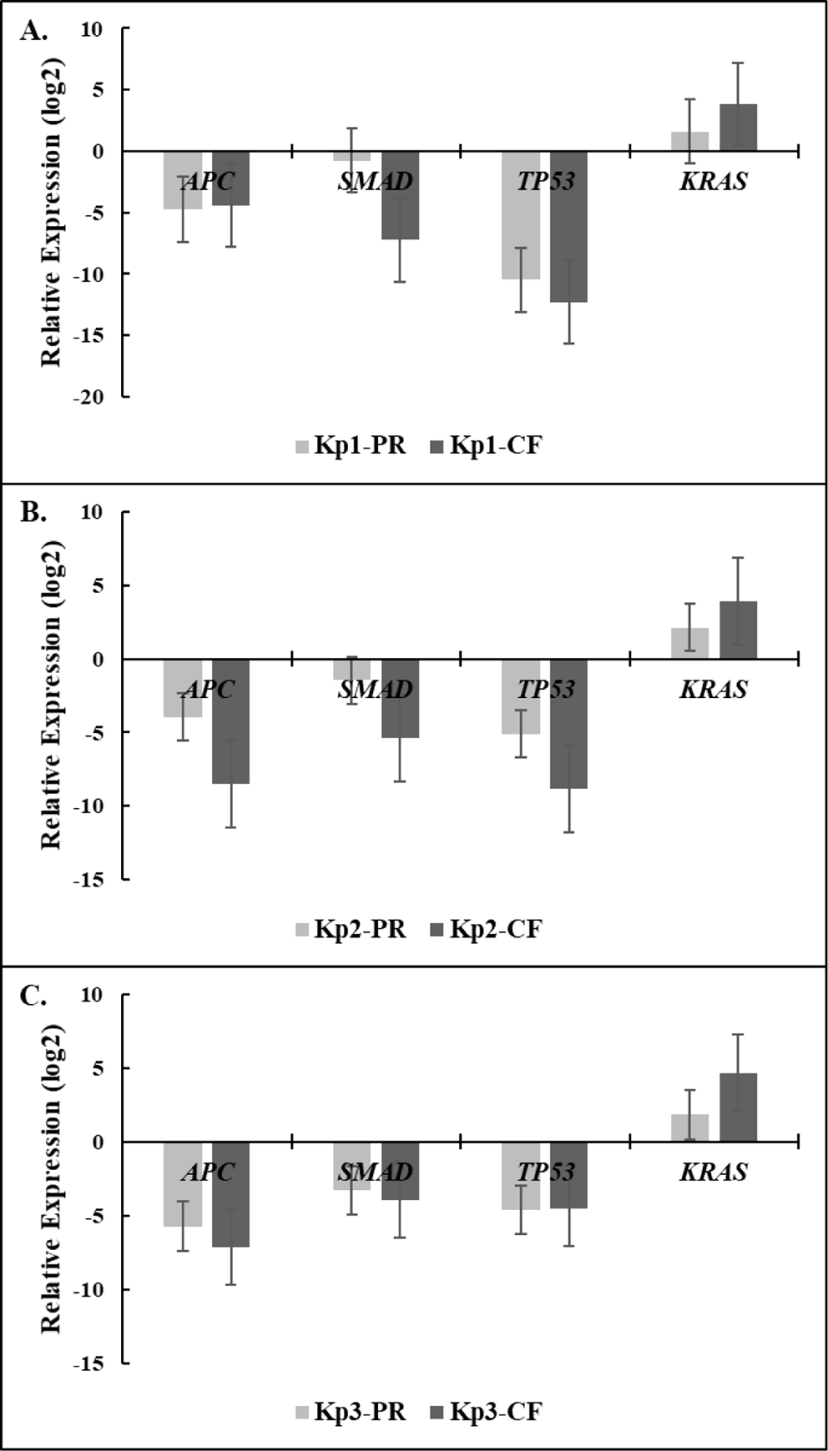
Relative expression of CRC-related genes in PCS-201-012 cells in response to the culture filtrates and bacterial proteins. (A) *Kp1*, (B) *Kp2*, and (C) *Kp3* strains. The values are presented as the log_2_ of the relative expression and represent the mean relative expression derived from biological triplicates. (PR: protein, CF: culture filtrate) (*p* < 0.01).

## 4 Conclusions

Cancer is a multifactorial disease, and many pathways are known to be important in its formation. In addition to many factors in the formation of cancer, some infectious factors are very effective to form cancer tissue. In this study, the relationship between CRC formation and the bacterial strains isolated from biopsy specimens of patients with CRC was investigated. In this context, the identities of the isolated bacterial strains were confirmed by 16S rRNA sequence analysis. As a result of isolations from 10 different CRC biopsy specimens, three *K. pneumoniae* strains were isolated. Previous studies have shown that many microorganisms (*Streptococcus* species, *Helicobacter pylori*, *E. faecalis*, *B. fragilis*, *Clostridium septicum*, and *E. coli*) together with *K. pneumoniae* are isolated from the colonic microbiota of colon cancer patients, and these microorganisms were found to play an important role in the formation and development of CRC.^32^ Bacterial microbiota induce colorectal carcinogenesis through various mechanisms. The primary suspected mechanisms are bacterial genotoxin production, microbial metabolism, modulation of host defenses, oxidative stress induction, inflammation, and antioxidant defense regulation. These mechanisms cause a variety of cellular effects and alterations in host defenses, involved in colorectal carcinogenesis, leading to genomic imbalance and epithelial cell proliferation.^33^

In this study, we aimed to assess the cytotoxic effects of *K. pneumoniae* against the normal human fibroblast PCS-201-012 cell model. Although many studies have shown that *K. pneumoniae* is associated with colorectal cancer over pyogenic liver abscess, as far as we know, there has been no study that compares the effects of their proteins and culture filtrates simultaneously. In light of this issue, the cytotoxic effects of the culture filtrates and total proteins of strains against PCS-201-012 cells have been characterized.

WST-8 and hemolysis tests were performed to observe the cytotoxic effects of the culture filtrates and bacterial proteins against a healthy fibroblast cell model. Both proteins and culture filtrates were found to reduce cell viability and IC_50_ values as shown in the WST-8 assay. According to the cell viability analysis of the bacterial proteins in PCS-201-012 cells, the IC_50_ values were found to be 46 ± 4.3 μg/mL for *Kp1*, 46 ± 7.1 μg/mL for *Kp2*, and 92 ± 5.9 μg/mL for *Kp3*. The viability of the cells exposed to the culture filtrates was found to be under 50% after the application of 100% and 50% culture filtrate. One of the important markers used in the determination of toxic or lytic byproducts produced by various strains is the determination of hemolytic activity. Therefore, the effects of the culture filtrates and bacterial proteins on human red blood cells (h-RBCs) were determined by the hemolysis test. Total proteins of *Kp1* (31.25, 46, 62.5 μg/mL), *Kp2* (31.25, 46, 62.5 μg/mL), and *Kp3* (62.5, 92, 125 μg/mL) strains and their culture filtrates of 100%, 50%, and 25% concentrations were applied to h-RBC. The results showed that the culture filtrates and bacterial proteins did not cause any detectable damage in human red blood cell membranes. It can be concluded that the lytic or toxic products in the culture filtrates and bacterial proteins of the strains are not in the range of concentrations that will cause hemolysis in the blood. On the other hand, the cytotoxicity observed in both the total protein and culture filtrate fractions of *K. pneumoniae* suggests a multifactorial mechanism, not limited to individual toxins. Previous research has shown that *K. pneumoniae* expresses a broad range of virulence-associated proteins, including outer membrane proteins, enzymes, and siderophore-related proteins, which can contribute to epithelial damage and immune modulation.^34,35^ In addition, culture filtrates may harbor metabolites and secreted components, including outer membrane vesicles from *K. pneumoniae*, which—although not traditionally classified as toxins—can induce inflammatory responses and display cytotoxic activity.^36^ As such, our findings indicate that the observed toxicity likely results from a synergistic effect of multiple proteinaceous components rather than a single virulence factor. Further studies involving proteomic and transcriptomic profiling are warranted to delineate the specific contributors to this effect.

To evaluate the apoptotic effects of the culture filtrates and bacterial proteins on PCS-201-012 cells, alterations in caspase-3 enzyme activity, mitochondrial membrane potential, and flow cytometry-based Annexin-V/PI double staining were examined. Caspase-3 is an important enzyme in apoptosis because of its role in coordinating the destruction of cellular structures. Incubation of PCS-201-012 cells with the culture filtrates (100% and 50%) demonstrated considerable induction of caspase-3 activity compared to untreated cells. However, we observed no increases in caspase-3 activity in the cells incubated with the bacterial proteins. These results showed that the bacterial proteins induce necrosis and not apoptosis. For further confirmation of these results, JC-1 analysis was performed. Among all treatment protocols, the 100% culture filtrate showed the highest fluorescence intensity in PCS-201-012 cells and led to 3.56-, 1.64-, and 1.96-fold increases in *Kp1*, *Kp2*, and *Kp3* strains as compared to the control group, respectively. The treatment with the bacterial proteins showed less mitochondrial depolarization with 1.30-, 1.90-, and 1.33-fold increases for *Kp1*, *Kp2*, and *Kp3* strains as compared to the control group, respectively. To corroborate the caspase-3 and mitochondrial membrane potential experiments, the percentage of cells undergoing apoptosis was analyzed by flow cytometry. The treatment with the culture filtrates increased the number of necrotic cells by nearly 9.1% compared to the bacterial proteins, confirming that all cell death after incubation with *Kp1*, *Kp2*, and *Kp3* strains occurred by necrosis.

To investigate the genetic alterations of PCS-201–012 cells after incubation with *Kp* strains, changes in the gene expression levels of four candidate genes (APC, SMAD, KRAS, TP53) were analyzed. The results showed that both the culture filtrates and bacterial proteins downregulated the tumor suppressor APC, SMAD, and TP53 genes, whereas they upregulated the oncogene KRAS gene in PCS-201-012 cells, confirming the functional involvement and tumorigenicity of the strains in colorectal cancer. These observed transcriptional changes suggest that secreted factors and protein components from *K. pneumoniae* may influence oncogenic signaling cascades. Notably, previous studies have demonstrated that bacterial outer membrane vesicles and other secreted effectors can modulate host gene expression and inflammatory responses, potentially contributing to tumorigenesis.^37,38^

In accordance with these results, it could be concluded that *K. pneumoniae* strains are capable of triggering cytotoxicity on human healthy cells, and these inflammations may promote all stages of tumorigenesis. Future studies are planned to include normal and cancerous human colonic epithelial cell lines (e.g., CCD 841 CoN, Caco-2, HT-29) to provide a more comprehensive understanding of host–pathogen interactions in CRC.
